# All-electrical recursive generation and time-domain mode division multiplexing of Hermite–Gaussian pulses for optical, THz and RF links

**DOI:** 10.1038/s41598-024-84267-6

**Published:** 2025-01-02

**Authors:** Xiaonan Yu, Janosch Meier, Paulomi Mandal, Mohamed I. Hosni, Abhinand Venugopalan, Lingfei Wang, Thomas Schneider

**Affiliations:** 1 THz-Photonics Group, Institut für Hochfrequenztechnik, Technische Universität Braunschweig, 38106 Braunschweig, Germany; 2 National and Local Joint Engineering Research Center of Space Optoelectronics Technology, Changchun University of Science and Technology, Changchun, 130022 China; 3 Optoelectronics Department, Military Technical College, Cairo, Egypt

**Keywords:** Optics and photonics, Electrical and electronic engineering

## Abstract

Space division multiplexing (SDM) with Hermite Gaussian (*HG*) modes, for instance, can significantly boost the transmission link capacity. However, SDM is not suitable in existing single mode fiber networks, and in long-distance wireless, microwave, THz or optical links, the far-field beam distribution may present a problem. Recently it has been demonstrated, that time domain *HG* modes can be employed to enhance the link capacity. However, implementing this method in wireless or fiber-based transmission systems is impractical due to the need for highly complex setups involving specialized lasers, wave shapers and other advanced devices. We propose a simple and fully electrical time-domain mode-division-multiplexing (TD-MDM) method based on the recursive generation of Hermite–Gaussian (HG) modes. It utilizes Gaussian pulse sequences, sawtooth signals, RF multipliers, adders, amplifiers, and Mach–Zehnder modulators for efficient multiplexing and demultiplexing. We show the time and bandwidth performance of 4 multiplexed orthogonal modes in transmitting 8 Gbps communication data (4 × 2 Gbit/s), demonstrating the feasibility of the recursive generation and multiplexing technique for TD-MDM with *HG* modes. The data rates were restricted by our experimental capabilities. With state-of-the-art equipment the method can easily be scaled to the terabit per second range.

## Introduction

**Fig. 1:**
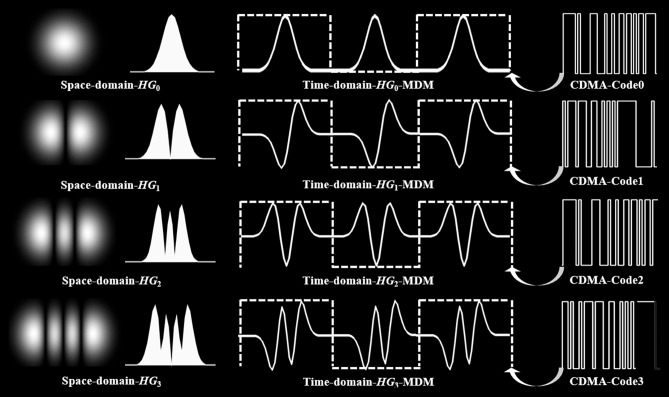
Changing from space to time domain of Hermite Gaussian Signals. 2D space distribution and cross section for *m* = 0, 1, 2, 3 (first and second column). The time domain *HG* pulse shapes are depicted in the third column with the solid lines. These pulse sequences are used for a sampling of the symbols (dashed lines) and added up for multiplexing. A comparison with CDMA (for one single symbol) is shown in the fourth column.

The requirements for the global information transmission capacity are increasing at a rate of 40% annually. So, in 20 years the needed capacity will be 1000 times higher than now^1^. To meet this challenge, all seven dimensions of multiplexing are used in modern communication systems, i.e. frequency, amplitude, phase, time, polarization, code and space, which has led to a considerable increase in the available transmission capacity^2–5^. Especially the successful transmission of linearly polarized (*LP*), Laguerre–Gaussian (*LG*) and Hermite–Gaussian (*HG*) modes in multicore optical fibers^6–8^, the gradual development of fan-in and fan-out devices^9,10^, and the application of massive MIMO in RF communications for space division multiplexing has arguably become the most successful method for increasing the capacity in communication systems. However, this space diversity introduces significant challenges for the link. In wired information transmission systems, this requires the use of specially developed multi-core or few-mode optical fibers, which increases the cost and complexity of the system. For wireless systems, it leads to higher demands on beam scanning and control, since the different modes result in distinct spatial far-field beam distributions^11,12^. For transceivers with an aperture of 100 mm and a divergence of 100 μrad, for instance, the spot size of an optical link in 1000 km distance would be 100 m (Fig. 1). This makes it impossible to collect all mode information^13,14^.

Recently, Nakazawa et al.^15–17^ have shown how the idea of orthogonal Hermite–Gaussian spatial modes can be converted into orthogonal time domain signals (see Fig. 1). In contrast to space domain multiplexing, the *N* orthogonal *HG* time-domain modes are multiplexed into one single time slot to increase the capacity of the transmission. This is similar to Code Division Multiple Access (CDMA), where baseband signals are multiplexed and demultiplexed using the orthogonality of pseudorandom codes. Time domain mode division multiplexing (TD-MDM) with *HG* modes thus shares the advantage of CDMA over time and frequency domain multiple access (TDMA and FDMA). It does not require additional time slots or frequency channels for additional users, which significantly increases spectral efficiency. However, while CDMA uses digital circuits with feedback shift registers to generate continuous spreading codes, the *HG* modes are generated as pulse sequences by analog circuits. This provides the *HG* modes with two key advantages: higher data rates due to possibly faster analog devices and improved transmission performance with lower duty cycles, enabling higher peak power and better signal-to-noise ratio (SNR), making it more suitable for high-speed optical communications.

A detailed analysis of the transmission properties of four multiplexed *HG* modes for a link distance of up to 450 km, modulation formats up to 64 QAM and bit rates up to 480 Gbit/s can be found in Ref.^17^. The setup in Ref.^17^ requires two optical comb generators, six wave shapers and high-speed arbitrary waveform generators. This makes the utilization of the method for optical links quite costly, power-hungry and almost impractical for RF or THz links. Here we show how such Hermite–Gaussian signals can be generated in the electrical domain based on a recursive process and the multiplexing and demultiplexing in the time domain based on a sampling process in a Mach–Zehnder modulator. The presented method offers much more flexibility, simplicity, and cost-effectiveness. Hence, it enables less power consumption in signal generation and transmission. Essentially it is a baseband pulse sequence generation and multiplexing method, making it suitable for any carrier and any channel. Therefore, it can be easily deployed in optical free-space or waveguide communication links and even in microwave or THz wireless systems.

Here, we present a recursive generation mechanism for the generation of Hermite–Gaussian signals, together with a simple multiplexing and demultiplexing technique based on the orthogonality of the modes. Additionally, we describe an *HG* transmitter in the electrical domain and a receiver based on orthogonal sampling^18^. In proof of concept experiments we demonstrate the time and bandwidth performance of our method for 4 TD-MDM modes with a data rate of 8 Gbit/s. This data rate was limited by our experimental capabilities and can easily be increased to much higher values.

## *HG* transceiver

### Mode generator

It is widely recognized that the *m*-order Hermite–Gaussian function is the solution of the Schrödinger equation:

1 $$\frac{{d^{2} }}{{dt^{2} }}HG_{m} \left( t \right) + \left( {\lambda_{m} - t^{2} } \right)HG_{m} \left( t \right) = 0,$$

with λ_m_ as the *m*-th eigenvalue. The solution can be expressed as:

2 $$HG_{m} \left( t \right) = H_{m} \left( t \right)e^{{ - t^{2} /2}} ,$$

where $H_{m} \left( t \right)$ is the Hermite polynomial function. As further discussed in the “Methods” section, the *m*-th order *HG* mode can be generated by the recursive equation:

3 $$H_{m} \left( t \right) = 2tH_{m - 1} \left( t \right) - 2\left( {m - 1} \right)H_{m - 2} \left( t \right).$$

Therefore, higher-order Hermite–Gaussian polynomials can be generated through multiplication, addition, and scalar operations of $2t$ with the previous and pre-previous order *HG* pulses $H_{m - 1} \left( t \right)$, and $H_{m - 2} \left( t \right)$, respectively. Therefore, Hermite–Gaussian pulse sequences can be achieved in a cascaded manner from the first mode by very simple electronic devices.

**Fig. 2:**
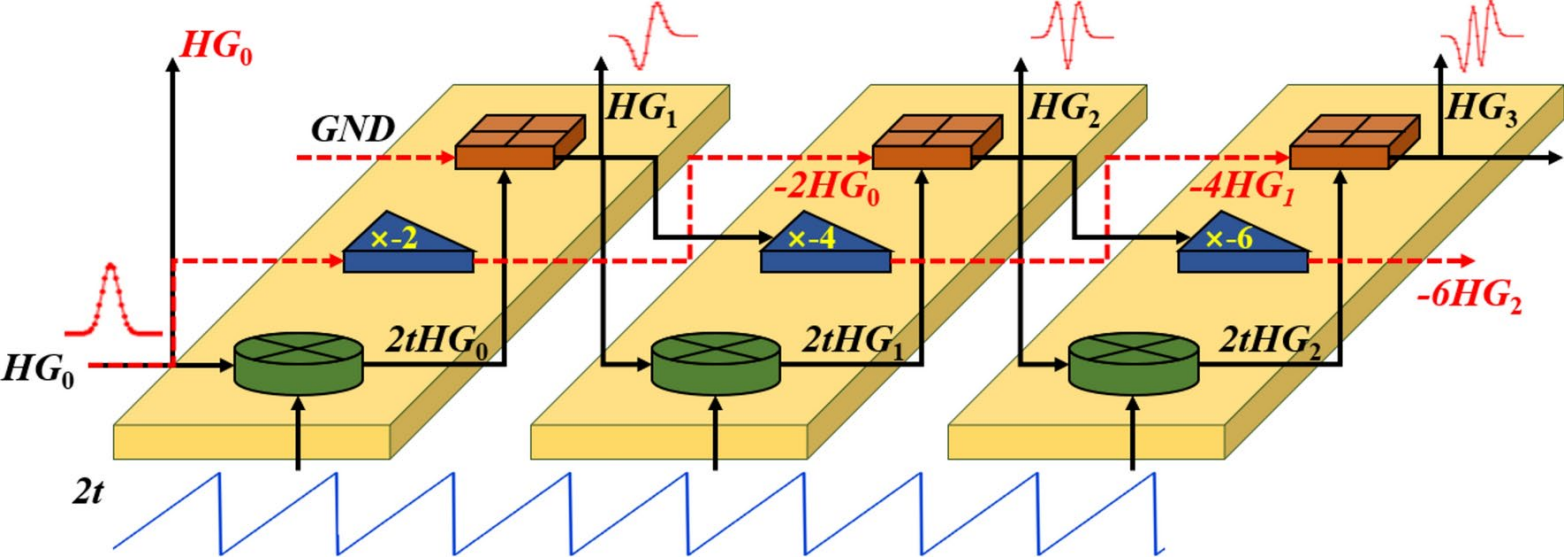
Recursive generation of 4 orders of time-domain Hermite–Gaussian pulse sequences with adders (⊞), amplifiers (⊳, − 2, − 4, − 6) and multipliers (⊗) from top to bottom.

Based on Eq. (3), we have designed a system for the generation of time-domain Hermite–Gaussian signals, as shown in Fig. 2. A zero-order electrical Hermite Gaussian signal (*HG*_0_) and a 2*t* sequence generated by a sawtooth signal generator are used as input signals. With these two basic signals, higher order *HG* pulse sequences can be generated according to Eq. (3). This process can be implemented by some basic RF devices such as multipliers, amplifiers, and adders. Each set of the *N* devices for the generation of the single *HG*_m_ mode has the same structure but different gains and bandwidths.

For the generation of the *HG*_0_ pulse sequence we are using the equation:

4 $$HG_{0} \left( n \right) = e^{{\left( {\left( {n - \frac{M}{2}} \right)/k} \right)^{2} /2}} ,$$

with *n* as a discrete variable, *M* as the number of sampling points over the pulse period and *k* as a frequency division coefficient which determines the duty cycle of the sequence. A Gaussian pulse sequence as described by Eq. (4) can be generated with an arbitrary waveform generator, the Gaussian filtering of a frequency comb or by some other means.

### Transceiver

**Fig. 3:**
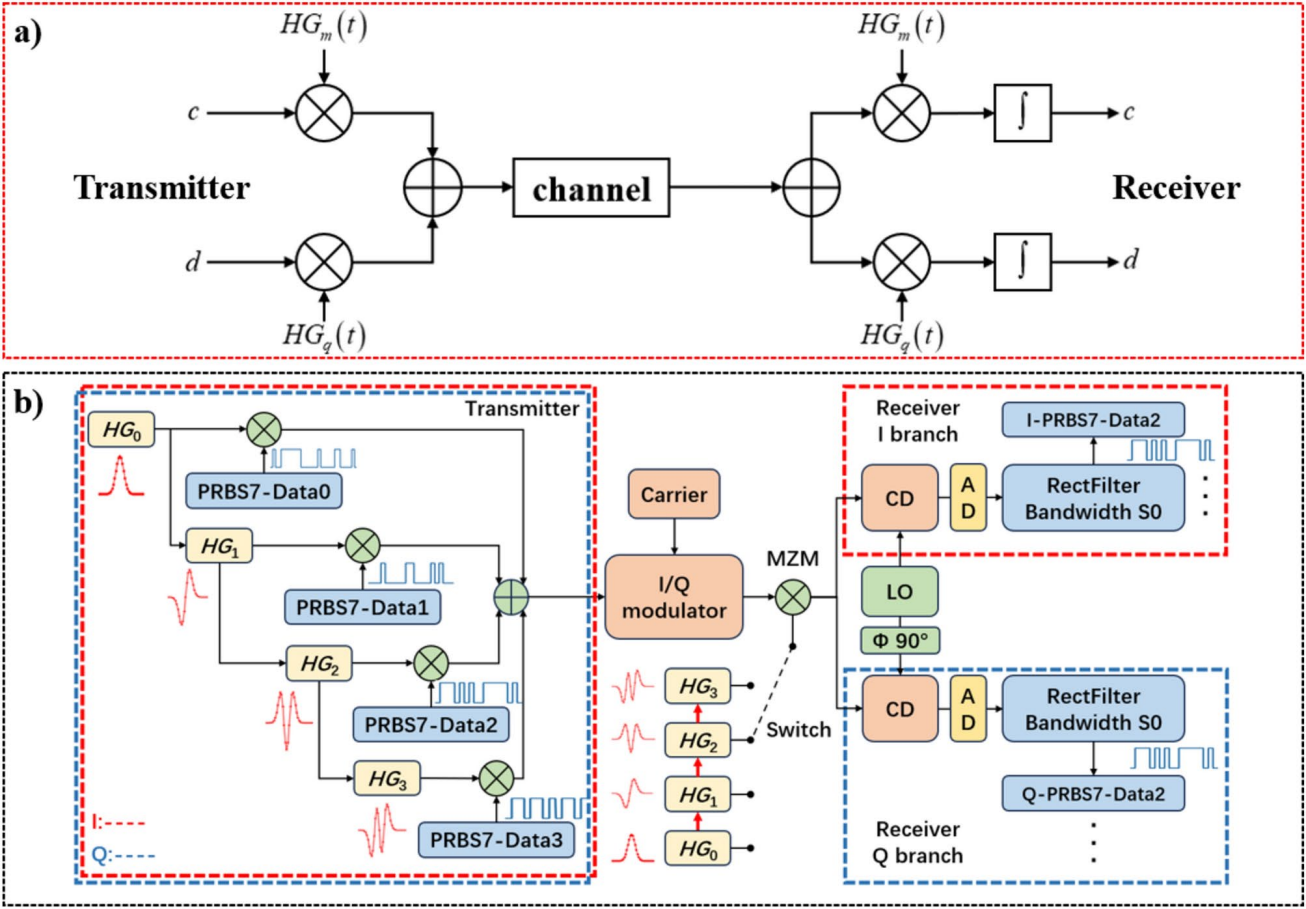
(**a**) Basic principle of multiplexing and demultiplexing with orthogonal *HG* pulse sequences. (**b**) Transmitter and receiver for the multiplexed transmission of 4 orders of *HG* pulse sequences. The red dashed box shows the required second transmitter and receiver for I/Q modulation. *CD* coherent detector, *AD* Analog to Digital Converter, *LO* local oscillator.

For the multiplexing and demultiplexing of the *HG* modes we exploit the orthogonality between them:

5 $$\int_{ - \infty }^{ + \infty } {HG_{m} \left( t \right)HG_{q} \left( t \right)dt} = 1,\quad if\;m = q,$$

6 $$\int_{ - \infty }^{ + \infty } {HG_{m} \left( t \right)HG_{q} \left( t \right)dt} = 0,\quad if\;m \ne q,$$

which leads to the schematic setup of the transmitter and receiver presented in Fig. 3a.

In Fig. 3a, c and d are the data channels. The data will be multiplied with the Hermite–Gaussian pulse sequences $HG_{q} \left( t \right)$ and $HG_{m} \left( t \right)$, with $q\ne m$, added and transmitted through the channel.

Based on this scheme, we have designed a *HG*-mode transceiver presented in Fig. 3b. The modulation with the data (Data 0, 1, 2 and 3) and the multiplexing of four *HG* modes (*N* = 4) is presented on the left side of Fig. 3b. The setup is similar to orthogonal sampling with sinc pulse sequences^19,20^. For each of the *N HG* modes a single branch is required. In each of the branches the symbols are multiplied with the *HG* sequence, which can be seen as a sampling process. After sampling, the signals from the four branches are summed up. Due to their orthogonality, they can be transmitted in the same time slot without mutual interference. The signal can then be modulated on an optical carrier by a Mach–Zehnder modulator, or any other carrier by the respective modulator. For I–Q signals, an additional sampling transmitter for the Q-branch is necessary (as shown with the red dashed block). The receiver is shown on the right side of Fig. 3b. A Mach–Zehnder modulator (MZM) is placed before the demodulator to multiply the locally generated orthogonal *HG* sequence with the received signal^21,22^. For the real-time demultiplexing of all *m* channels, the receiver requires *m* parallel branches. Alternatively, by switching between the different *HG* modes, the respective signal can be demultiplexed. Please note that according to Fig. 3b, only the multiplication between the signal and the unmodulated *HG* sequence is necessary. Thus, even for I–Q signals a single MZM is sufficient for demultiplexing. The rectangular filter in the baseband accomplishes the integration in Fig. 3b and doesn’t have to be ideally rectangular. The bandwidth of that filter corresponds to the baseband bandwidth of the single PRBS data signal. Therefore, already the coherent detector as well as all following signal processing does not need the bandwidth of the pulse sequences or that of the multiplexed signal, just the bandwidth of the data signal is sufficient. If the coherent detector has the baseband bandwidth of the signal, it accomplishes the integration and the filter is not necessary. For lower bandwidths analog to digital converters (ADC) a better signal to noise and distortion ratio and correspondingly effective number of bits can be expected, which may lead to a lower power consumption for the following digital signal processing. The demultiplexing or multiplication of the multiplexed signal with the *HG* pulse sequence can as well be carried out in the baseband by electronic signal processing. However, in this case the coherent detector and the analog to digital converter needs the full bandwidth of the multiplexed signal.

With increasing order, the bandwidth of the HG modes increases (please see the “Methods” section). This increasing bandwidth for the higher order modes directly affects the recursive generation, which will be measured and analyzed in the subsequent simulations and experiments.

## Results

### Simulation results

**Fig. 4:**
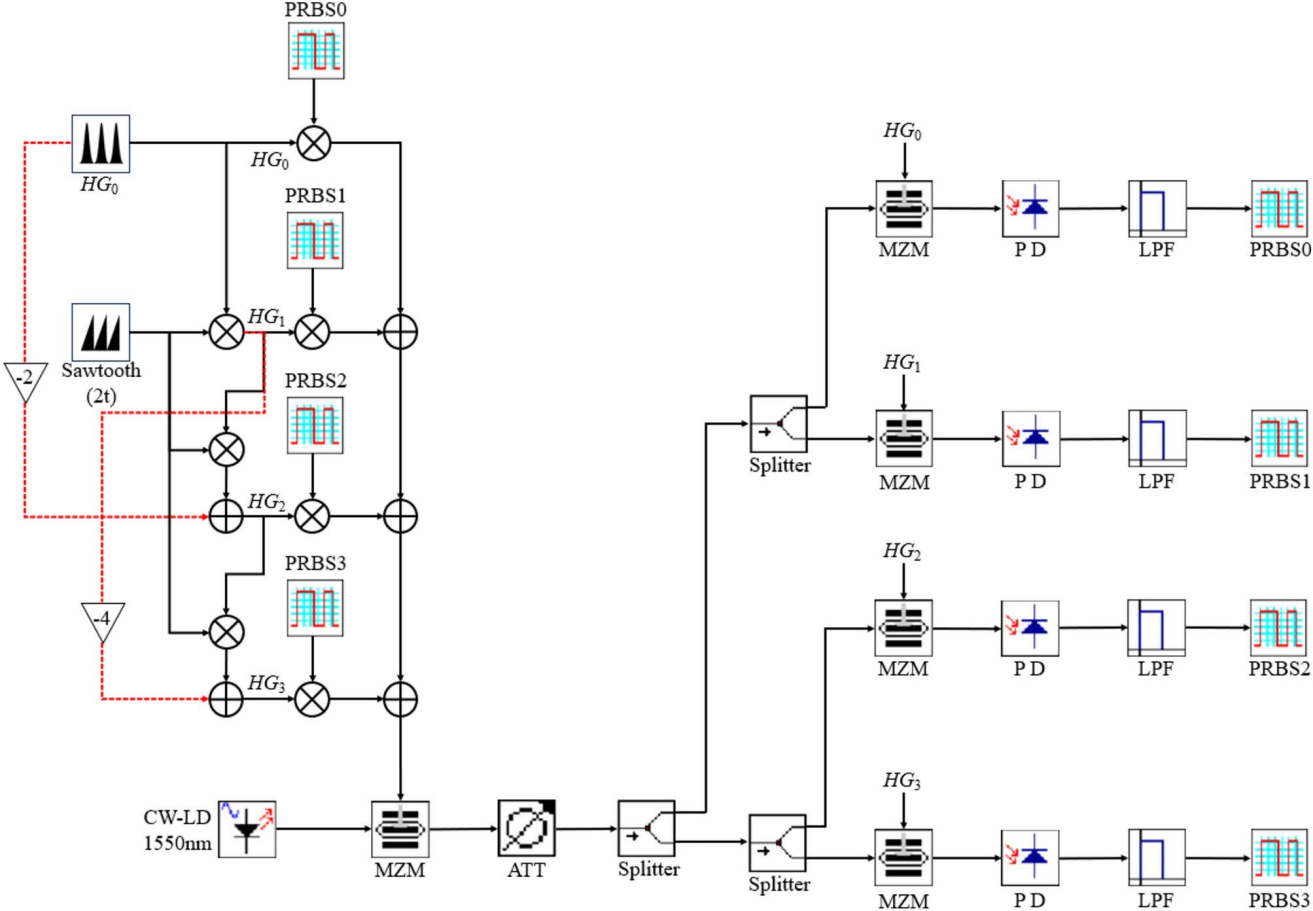
Simulation setup of the four-mode *HG* multiplexing transmission link. *ATT* variable attenuator, *LPF* low pass filter, *CW-LD* continuous wave photodiode, *PD* photodiode, *MZM* Mach–Zehnder modulator.

To analyze the performance of the *HG* multiplexed transmission, generated recursively from the *HG*_0_ signal, we conducted a series of simulation studies with the Opti-System software package. The simulation setup can be seen in Fig. 4.

Four *HG* modes were recursively generated from a 2 GHz *HG*_0_ sequence with a repetition rate of 500 ps and a sawtooth signal. The four modes were subsequently multiplied by different pseudo-random bit sequences (PRBS-7) and summed up to obtain the multiplexed signal. In the receiver, the multiplexed signal was multiplied with the corresponding unmodulated *HG* sequence using an MZM, and then down-converted to the baseband by a coherent detector driven with a local oscillator (LO) wave. In the baseband a rectangular filter with the bandwidth of the PRBS data was incorporated to recover the PRBS sequence, which was analyzed with an eye diagram and bit error rate tester.

**Fig. 5:**
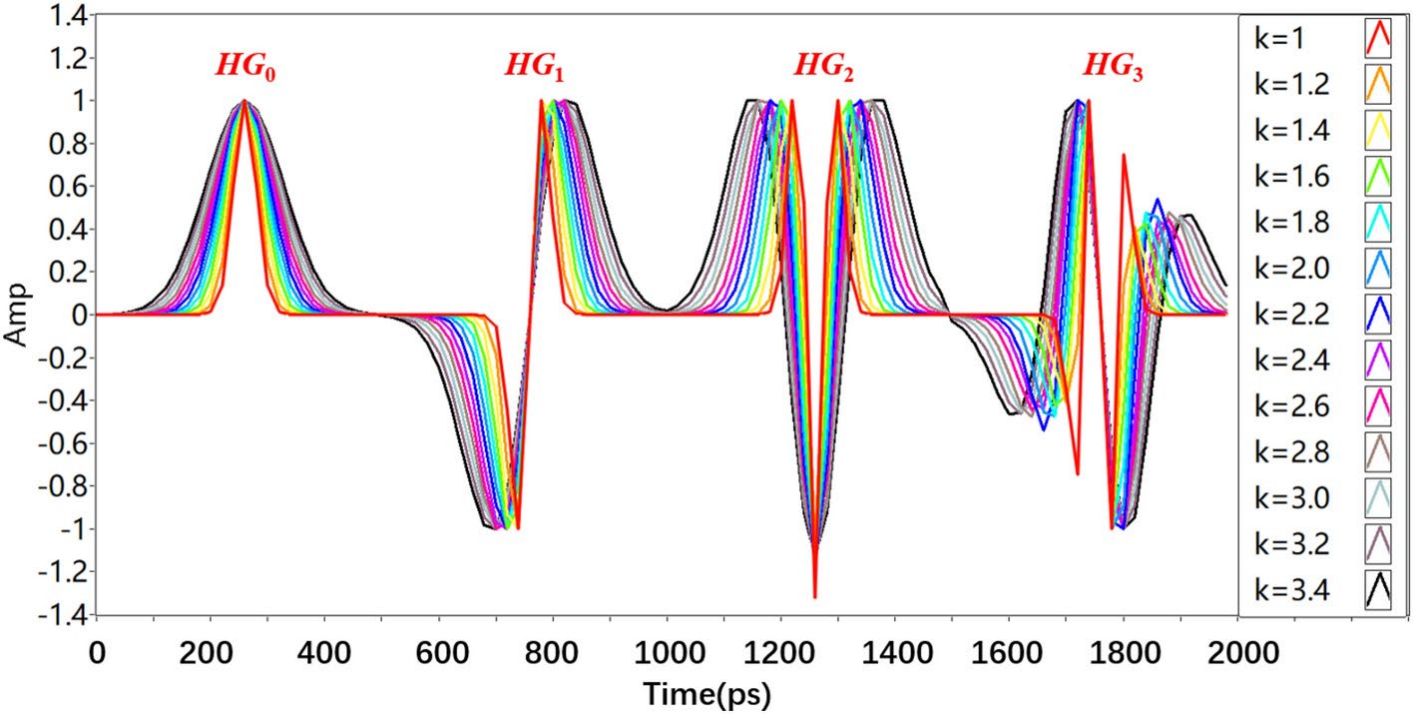
Relation between pulse width and repetition rate (defined by the *k* parameter) for *HG*_0_ (first, from left to right) and the recursively generated pulses (*HG*_1_ second, *HG*_2_ third and *HG*_3_ fourth).

In first simulations we were examining the impact of the relation between pulse width and repetition rate (defined by the *k* parameter for the *HG*_0_ sequence in Eq. 4) on the transmission performance. The recursively generated *HG* sequences (*HG*_0_–*HG*_3_) are displayed in Fig. 5 for a 500 ps time interval, a sampling rate of 50 GS/s and *k* = 1 to *k* = 3.4 with an increment of 0.2.

As can be seen from Fig. 5, the time duration of the higher order *HG* pulses increases. Since all these pulse sequences are multiplexed together, and the higher orders are recursively generated from *HG*_0_, it is important to optimize the duration of *HG*_0_ in a manner that the symbol rate is as high as possible, while the bit error rate (BER) for all 4 multiplexed modes is as low as possible.

**Fig. 6:**
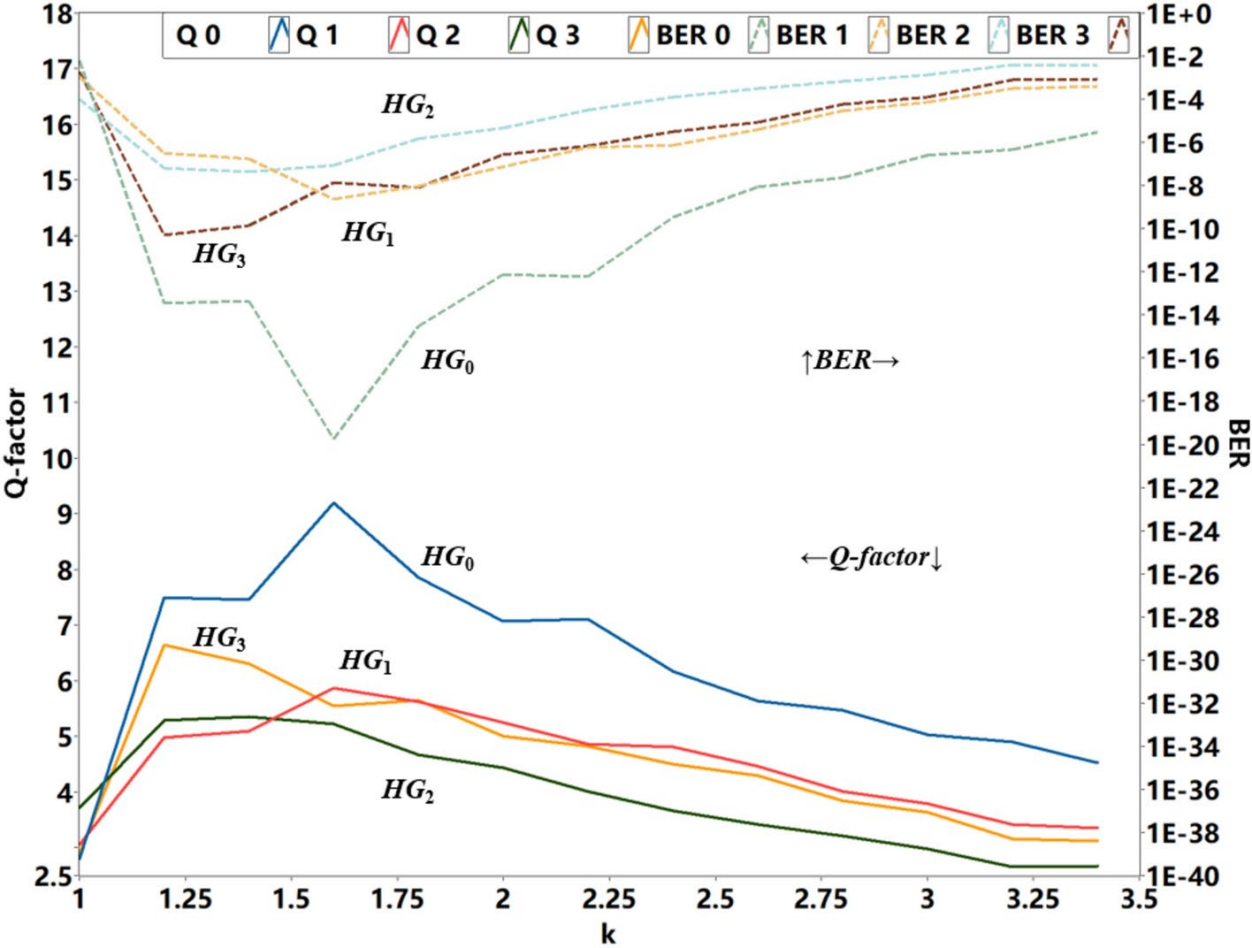
Relationship between the Q-factor (solid lines and left y axis) as well as BER (dashed lines and right y axis) and the pulse width of *HG*_0_ and the subsequently generated higher modes, as defined by *k*.

Therefore, we have simulated the transmission performance in dependence on the *k* factor for the whole setup in Fig. 4 and a very low signal to noise ratio (SNR). In the simulation we have multiplexed 4 modulated *HG* modes for different *k* values (from *k* = 1 to *k* = 3.4 in increments of 0.2) and analyzed the eye diagrams of the demultiplexed and received signal, the specific Q-factors and BERs resulting from these eye diagrams are shown in Fig. 6. All four modes show the maximum Q factor and correspondingly the lowest BER for $k\approx 1.6$.

Consequently, the best results can be seen if the relation between pulse width and repetition rate are not too small and not too wide. If the pulse width of *HG*_0_ is too wide, higher order modes, generated from the first order, exceed the duration between two consecutive time slots, which may lead to an inter-symbol-interference. If the pulses are too small, the bandwidth requirements increase, potentially leading to loss of frequency information and breaking orthogonality. Therefore, we have chosen *k* = 1.6 for the experiments. Please note that this may vary, if another number of modes are multiplexed.

If we assume that 99% of the total energy is contained in the defined duration, for *k* = 1.6, the corresponding time durations for *HG*_*0*_ to *HG*_*3*_ are 198 ps, 236 ps, 268 ps and 274 ps, respectively. According to Ref.^23^, the time-bandwidth product of *HG* signals is:

7 $$\Delta T_{m} \Delta F_{m} \cong 1.27m + 0.64,$$

where $\Delta T_{m}$ and $\Delta F_{m}$ are the time duration and bandwidth of the *HG*_m_ modes, and *m* is their order. Therefore, it follows for the bandwidth of the *HG* symbols: *HG*_0_ = 3.23 GHz, *HG*_1_ = 8.09 GHz, *HG*_2_ = 11.86 GHz and *HG*_3_ = 16.24 GHz. Thus, the highest bandwidth requirement for the devices used to generate the *HG* modes via the recursive method is around 16 GHz. As described in Ref.^23^, the bandwidth requirement is 0.64 times that of traditional OFDM signals. However, due to our demultiplexing technique, for the electronics in the receiver only the bandwidth of the single data signal (2 GHz) is required.

### Experimental results

Please note that a detailed analysis of the transmission properties of four multiplexed *HG* modes in an optical link can be found in Ref.^17^. The focus of this paper is the recursive generation as well as multiplexing and demultiplexing of the modes. Therefore, only proof of concept experiments with a quite small fiber length of a few meters have been conducted. Additionally, we only have two AWGs in the lab. Since one was required for the signal generation and the other for the demultiplexing, higher order modulation formats and especially I-Q modulations could not be tested. This can be circumvented if the demultiplexing is carried out in the baseband.

**Fig. 7:**
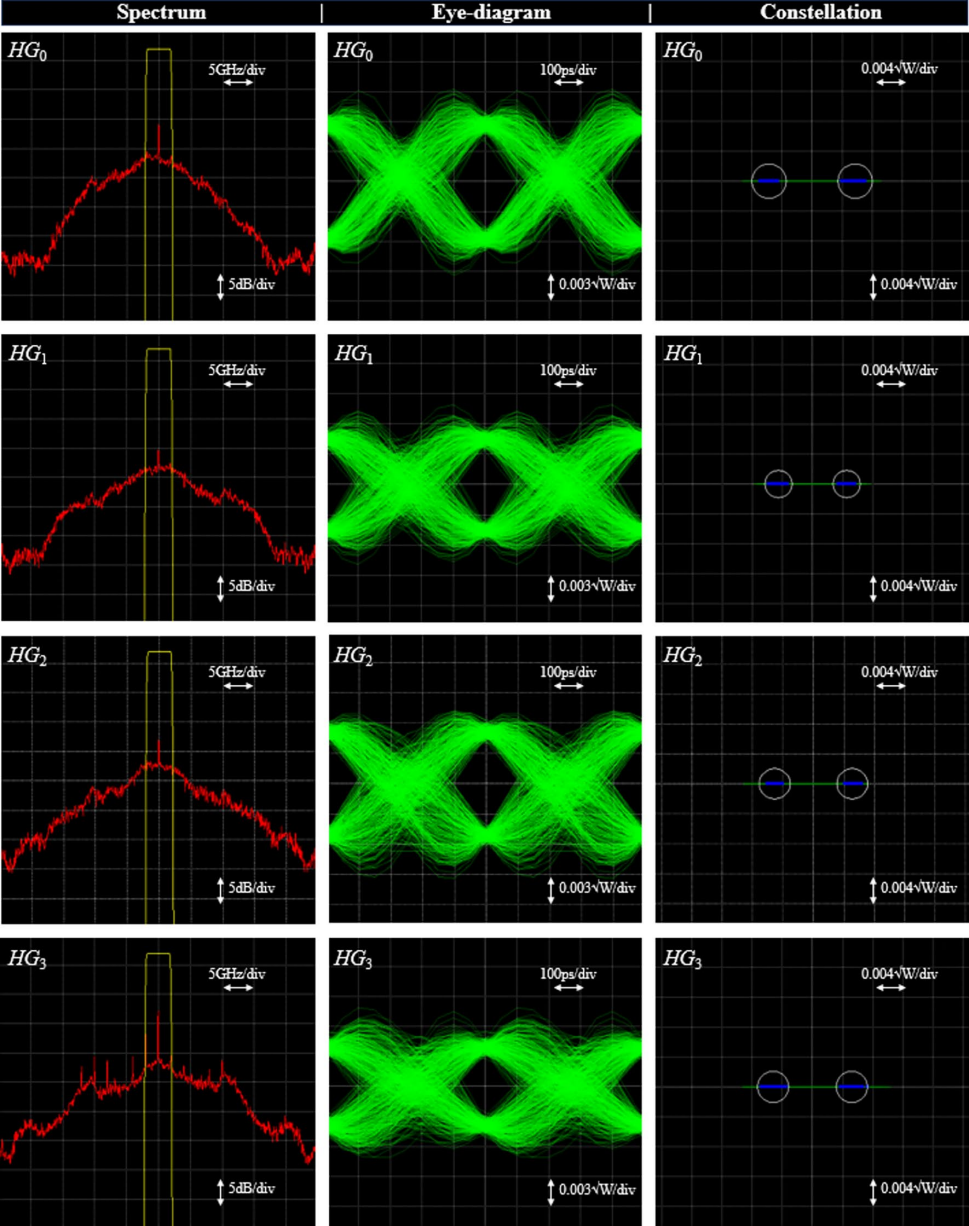
Spectrum, eye and constellation diagrams of the demultiplexed and received *HG*_0_, *HG*_1_, *HG*_2_ and *HG*_3_ mode for a bit rate of 4 × 2 Gbd = 8 Gbd. The Q factor for *HG*_0_ is 21.7 dB, *HG*_1_ 21.2 dB, *HG*_2_ 21.1 dB and for the *HG*_3_ mode 19.6 dB. There was no error in 20,000 transmitted bits. The rectangular function in the spectra represents the part that is filtered. The used baseband filter has half of this bandwidth.

The measured and normalized eye diagrams for the four modes with *k* = 1.6 are shown in Fig. 7. As can be seen, the very simple recursive generation of the *HG* modes as well as the simple modulation by sampling and the demultiplexing in a single MZM lead to very good results. Since we have used a higher SNR and a different modulation format than for the simulation in Fig. 6, the achieved Q-factor is with around 20 dB much higher for all modes.

Please note that the detector and signal processing just need the bandwidth of the data signal in the single mode (2 GHz) and not that of the pulses or the multiplexed signal (16 GHz). Therefore, the ADC bandwidth is reduced which may increase the signal to noise and distortion ratio and the effective number of bits, as investigated for sinc-pulse sequences in Ref.^24^.

## Discussion

We have presented a method for the very simple recursive generation, multiplexing and demultiplexing of subsequent multi-order *HG* modes. The method just requires a Gaussian pulse sequence and a sawtooth wave and can generate, modulate and multiplex any order of *HG* modes by software, electronics or dedicated hardware. Modulation and multiplexing are based on sampling the symbol with the respective pulse of the *HG* sequence and adding them together. Demultiplexing can be carried out with a single intensity modulator or in the baseband. If a modulator is used for demultiplexing, the bandwidth of the required detector and subsequent electronics corresponds to that of the single mode in the multiplexed sequence. Through simulations we have optimized the relation between pulse width and repetition rate for the recursive generation and we have presented proof of concept experiments for a 4 × 2 GBd signal transmission. The data rate was limited by the available instrumentation and equipment. Since the method only relies on fundamental analog computational mechanisms, it can be implemented on a chip. Therefore, it has the potential to achieve data rates, only restricted by the bandwidth of the electronics. Compared to other realizations^15–17^, the proposed technique effectively reduces complexity and cost, providing a simple and feasible multiplexing method for optical links, free-space optical communication as well as microwave and THz communication.

## Methods

### Principle of generation

As discussed in Ref.^17^, Hermite polynomials can be generated based on the McLaurin expansion. For even numbers of *m*, the *m*-th polynomial is:

8 $$\begin{aligned} H_{m} \left( t \right) = & m!\sum\limits_{n = 0}^{m/2} {\frac{{\left( { - 1} \right)^{n} }}{{n!\left( {m - 2n} \right)!}}\left( {2t} \right)^{m - 2n} } \\ { = } & 2^{m} t^{m} - \frac{{2^{m - 2} m!}}{{\left( {m - 2} \right)!}}t^{m - 2} + \frac{{2^{m - 4} m!}}{{2!\left( {m - 4} \right)!}}t^{m - 4} \\ {\kern 1pt} & - \frac{{2^{m - 6} m!}}{{3!\left( {m - 6} \right)!}}t^{m - 6} + \cdots + \left( { - 1} \right)^{m/2} \frac{m!}{{\left( \frac{m}{2} \right)!}}. \\ \end{aligned}$$

When *m* is odd, Eq. (8) becomes:

9 $$\begin{aligned} H_{m} \left( t \right) = & m!\sum\limits_{n = 0}^{{{{\left( {m - 1} \right)} \mathord{\left/ {\vphantom {{\left( {m - 1} \right)} 2}} \right. \kern-0pt} 2}}} {\frac{{\left( { - 1} \right)^{n} }}{{n!\left( {m - 2n} \right)!}}\left( {2t} \right)^{m - 2n} } \\ { = } & 2^{m} t^{m} - \frac{{2^{m - 2} m!}}{{\left( {m - 2} \right)!}}t^{m - 2} + \frac{{2^{m - 4} m!}}{{2!\left( {m - 4} \right)!}}t^{m - 4} \\ {\kern 1pt} & - \frac{{2^{m - 6} m!}}{{3!\left( {m - 6} \right)!}}t^{m - 6} + \cdots + \left( { - 1} \right)^{{\frac{m - 1}{2}}} \frac{2 \cdot m!}{{\left( {\frac{m - 1}{2}} \right)!}}t. \\ \end{aligned}$$

So, the first five polynomials can be written as:

10 $$\begin{gathered} H_{0} \left( t \right) = 1, \hfill \\ H_{1} \left( t \right) = 2t, \hfill \\ H_{2} \left( t \right) = 4t^{2} - 2, \hfill \\ H_{3} \left( t \right) = 8t^{2} - 12t, \hfill \\ H_{4} \left( t \right) = 16t^{4} - 48t^{2} + 12. \hfill \\ \end{gathered}$$

From Eq. (10) it can be deduced:

11 $$\begin{gathered} H_{0} \left( t \right) = 1, \hfill \\ H_{1} \left( t \right) = 2tH_{0} \left( t \right), \hfill \\ H_{2} \left( t \right) = 2tH_{1} \left( t \right) - 2H_{0} \left( t \right), \hfill \\ H_{3} \left( t \right) = 2tH_{2} \left( t \right) - 4H_{1} \left( t \right), \hfill \\ H_{4} \left( t \right) = 2tH_{3} \left( t \right) - 6H_{2} \left( t \right). \hfill \\ \end{gathered}$$

Leading to the Recursive Eq. (3).

Since the Fourier transform of the Schrödinger equation (Eq. 2) remains the Schrödinger equation, the Fourier transform of the *HG* signal is also an *HG* function with a constant factor:

12 $$HG_{m} \left( \omega \right) = \left( { - i} \right)^{m} \sqrt {2\pi } H_{m} \left( \omega \right)e^{{ - \omega^{2} /2}} ,$$

thus

13 $$\begin{gathered} HG_{0} \left( \omega \right) = \sqrt {2\pi } e^{{ - \omega^{2} /2}} , \hfill \\ HG_{1} \left( \omega \right) = - 2i\sqrt {2\pi } \omega e^{{ - \omega^{2} /2}} , \hfill \\ HG_{2} \left( \omega \right) = - 4\sqrt {2\pi } \omega^{2} e^{{ - \omega^{2} /2}} + 2\sqrt {2\pi } e^{{ - \omega^{2} /2}} . \hfill \\ \end{gathered}$$

## Multiplexing and demultiplexing

If two channels are multiplexed, the combined signal can be expressed as:

14 $$s\left( t \right) = c \cdot HG_{m} \left( t \right) + d \cdot HG_{q} \left( t \right).$$

In the receiver, the whole signal is power-split into *m*-branches (here *m* = 2), please see Fig. 3a. In the upper branch the signal is multiplied with the unmodulated pulse-sequence $HG_{m} \left( t \right)$, and in the lower branch with $HG_{q} \left( t \right)$. After integration in the detector, the data signal is retrieved in the baseband.

The equations for the demultiplexing are:

15 $$\begin{aligned} m\left( t \right) = & \int_{ - \infty }^{ + \infty } {\left( {\left( {c \cdot HG_{m} \left( t \right) + d \cdot HG_{q} \left( t \right)} \right) \cdot HG_{m} \left( t \right)} \right)dt} \\ = & \int_{ - \infty }^{ + \infty } {\left( {c \cdot HG_{m} \left( t \right)HG_{m} \left( t \right)} \right)dt} \\ & + \int_{ - \infty }^{ + \infty } {\left( {d \cdot HG_{q} \left( t \right)HG_{m} \left( t \right)} \right)dt} \\ = & \alpha \cdot c, \\ \end{aligned}$$

16 $$\begin{aligned} q\left( t \right) = & \int_{ - \infty }^{ + \infty } {\left( {\left( {c \cdot HG_{m} \left( t \right) + d \cdot HG_{q} \left( t \right)} \right) \cdot HG_{q} \left( t \right)} \right)dt} \\ = & \int_{ - \infty }^{ + \infty } {\left( {c \cdot HG_{m} \left( t \right)HG_{q} \left( t \right)} \right)dt} \\ & + \int_{ - \infty }^{ + \infty } {\left( {d \cdot HG_{q} \left( t \right)HG_{q} \left( t \right)} \right)dt} \\ = & \beta \cdot d, \\ \end{aligned}$$

where α and β indicate the amplitude of the signal and the conversion efficiency of the receiver. The orthogonality is given for an unlimited integration of the time functions, but practically we just integrate over the pulse duration by a low-pass filter. In the experimental part we have shown that this is sufficient for the practical differentiation of the various modes.

## Experimental setup

**Fig. 8:**
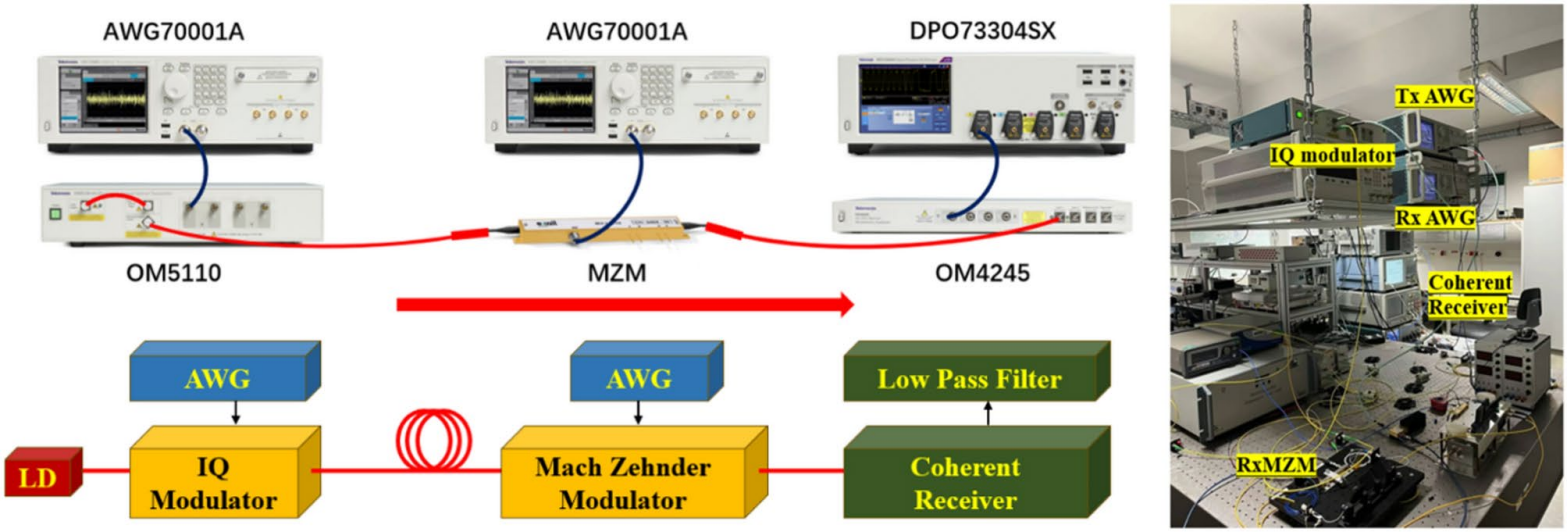
Experimental configuration for the transmission of 4 multiplexed *HG* modes, modulated with 4 × 2 GBd PRPS-7 data.

Based on the above description the experimental setup illustrated in Fig. 8 is used.

A 1550 nm laser diode (LD) serves as the carrier source. The *HG*_*0*_ to *HG*_*3*_ modulated and multiplexed modes are electronically generated by the recursive method, as described in Figs. 2 and 3, in an arbitrary waveform generator (Tektronix AWG70001A) and modulated onto the laser carrier through a modulator (Tektronix OM5110). After amplification and bandpass filtering to reduce the amplified spontaneous emission noise (not shown in Fig. 8), the signal is fiber connected to the receiver.

At the receiver, a single Mach–Zehnder modulator (MZM) is used for the demultiplexing. Another AWG generates the unmodulated *HG*_0_-*HG*_3_ sequences which are used for driving the MZM. A coherent detector and a real time oscilloscope (Tektronix OM4245 and DPO733304SX), detect the demultiplexed signals. A digital software filter of 2 GHz recovers the baseband signal.

## Data Availability

The datasets generated during and/or analysed for the current study are available from the corresponding author on reasonable request.
